# Identification of Mild Cognitive Impairment From Speech in Swedish Using Deep Sequential Neural Networks

**DOI:** 10.3389/fneur.2018.00975

**Published:** 2018-11-15

**Authors:** Charalambos Themistocleous, Marie Eckerström, Dimitrios Kokkinakis

**Affiliations:** ^1^The Swedish Language Bank, Department of Swedish, University of Gothenburg, Gothenburg, Sweden; ^2^Department of Neurology, Johns Hopkins University, School of Medicine, Baltimore, MD, United States; ^3^Department of Psychiatry and Neurochemistry, Institute of Neuroscience and Physiology, University of Gothenburg, Gothenburg, Sweden

**Keywords:** speech production, vowels, prosody, neural network, machine learning, dementia, MCI

## Abstract

While people with mild cognitive impairment (MCI) portray noticeably incipient memory difficulty in remembering events and situations along with problems in decision making, planning, and finding their way in familiar environments, detailed neuropsychological assessments also indicate deficits in language performance. To this day, there is no cure for dementia but early-stage treatment can delay the progression of MCI; thus, the development of valid tools for identifying early cognitive changes is of great importance. In this study, we provide an automated machine learning method, using Deep Neural Network Architectures, that aims to identify MCI. Speech materials were obtained using a reading task during evaluation sessions, as part of the Gothenburg MCI research study. Measures of vowel duration, vowel formants (*F*1 to *F*5), and fundamental frequency were calculated from speech signals. To learn the acoustic characteristics associated with MCI vs. healthy controls, we have trained and evaluated ten Deep Neural Network Architectures and measured how accurately they can diagnose participants that are unknown to the model. We evaluated the models using two evaluation tasks: a 5-fold crossvalidation and by splitting the data into 90% training and 10% evaluation set. The findings suggest first, that the acoustic features provide significant information for the identification of MCI; second, the best Deep Neural Network Architectures can classify MCI and healthy controls with high classification accuracy (*M* = 83%); and third, the model has the potential to offer higher accuracy than 84% if trained with more data (cf., *SD*≈15%). The Deep Neural Network Architecture proposed here constitutes a method that contributes to the early diagnosis of cognitive decline, quantify the progression of the condition, and enable suitable therapeutics.

## 1. Introduction

Individuals with mild cognitive impairment (MCI) portray a noticeable memory difficulty in remembering events and situations along with problems in decision making, planning, interpreting instructions, and orientation (1–5). These cognitive problems become frequent and more severe compared to the cognitive decline in normal aging (see also 6, 7). As the MCI progresses, MCI individuals face a higher risk of developing Alzheimer's Disease (AD).

In search of less strenuous and non-invasive techniques for assessing MCI, currently, there has been substantial interest on the role of speech and language and its potentials as markers of MCI. Language impairment in AD is well established (e.g., 8–10) and can be evaluated by using assessments, such as naming tests (11), discourse (12–14), verbal fluency tests (e.g., 15), complexity measures, such as phonemes per word, phone entropy, verbal fluency, and word recall (8–10, 13, 16–22). Findings with respect to syntax and phonology have been inconsistent though (for a discussion on the role of syntax in MCI, see 23). Also, many studies explored the interactions of language and other predictors from imaging, biomarkers etc., in dementia (24–30). The fact that language impairment occurs early and commonly in the progression of AD, motivated many researchers to identify markers of language impairment in MCI. For example, Manouilidou et al. (31) showed that while MCI individuals preserve morphological rule knowledge, they face processing difficulties of pseudo-words (for a discussion and review of current studies, see 32, 33). As there is only a handful of studies on the acoustic properties of MCI speech (e.g., 30, 34), more research on speech acoustics is required to gain a better understanding of how MCI speech differs from that of healthy controls.

The development of automated machine learning models that can *learn* the characteristics of MCI and provide an early and accurate identification of MCI is of utmost importance for two main reasons: First, an early identification can enable multidomain life style interventions and/or pharmacological treatments at the MCI stage, or even earlier, which can potentially delay or might even prevent the development of AD and other types of dementia (5, 35). Second, the early identification, will provide time to patients and their families to make decisions about their care, family issues, and legal concerns (5).

The aim of this study is to provide an automated method that can identify MCI individuals and distinguish them from healthy controls using acoustic information. Specifically, in this study, we provide an automated machine learning method using Deep Neural Network Architectures that identifies individuals with MCI from healthy controls. We demonstrate its performance by using data from Swedish. Specifically, 55 Swedish participants, 30 healthy controls and 25 MCI, were instructed by a clinician to read a short passage, consisting of 144 words, as part of their evaluation. Reading tasks are being employed extensively in research because they provide rich linguistic data without straining the participants (36). Also, they have the advantage that they are restrictive with respect to the segmental environment of vowels and consonants, which is the same for all participants. Next, the speech material was transcribed and segmented into vowels and consonants. From the segmented material, we measured vowel *F*1−*F*5 formant frequencies, *F*0, and duration. Vowel formants are a range of vowel frequency peaks in the sound spectrum. Formant frequencies are the primary acoustic correlates for the production of vowels. *F*1 and *F*2 usually suffice for the identification of vowels in most languages but higher order formant frequencies can provide information about the social—such as the age, gender, and dialect—and physiological properties of speakers (37–39). In Swedish, *F*3 also contributes to the distinction of rounded and unrounded vowels (40). *F*0 is the acoustic correlate of intonation. Speakers vary the *F*0 of their utterances to produce various melodic patterns, such as when emphasizing parts of the utterance, asking questions, giving commands, etc. *F*0 (e.g., mean *F*0, *F*0 minimum and maximum) is found to be lower in individuals with depression (41, 42). In addition to frequency measurements, we measured vowel duration.

For the classification task, we have evaluated several Deep Neural Network Architectures based on Multilayer Perceptrons (MLP). MLPs are a type of sequential, Feed-Forward Neural Network, which when trained on a dataset, can learn a non-linear function approximator for the classification of MCI and healthy participant:

(1) $$\begin{array}{r} f\left(\cdot \right):R^{m}\to R^{o} \end{array}$$

where *m* is the number of dimensions for input and *o* is the number of dimensions for output. Given a set of vowel features *X* = *x*_1_, *x*_2_, …, *x*_*m*_ and a target *y*; namely, an array of values determining the condition of the participant (healthy controls vs. MCI), the neural network can learn the classification function. The advantage of this type of network for our data is that it can learn non-linear structures.

## 2. Methodology

In this section, we describe the development of the dataset and the structure of the predictors.

### 2.1. Speech materials

Participants for this study were recruited from the Gothenburg MCI study, which is a large clinically based longitudinal study on mild cognitive impairment (5). This study aims to increase the nosological knowledge that will enable rational trials in AD and other types of dementia. It also includes longitudinal in-depth phenotyping of patients with different forms and degrees of cognitive impairment using neuropsychological, neuroimaging, and neurochemical tools (5). Speech recordings were conducted as part of the additional assessment tests that conduced for the purposes of the Riksbankens Jubileumsfond – The Swedish Foundation for Humanities & Social Sciences “Linguistic and extra-linguistic parameters for early detection of cognitive impairment” research grant (NHS 14-1761:1).

### 2.2. Participants

The recordings were conducted in an isolated environment at the University of Gothenburg. Thirty healthy controls and 25 MCI—between 55 and 79 years old (*M* = 69, *SD* = 6.4) participated in the study (see Table 1). The two groups did not differ with respect to age [*t*_(52.72)_ = −1.8178, *p* = *n*.*s*.] and gender (*W* = 1567.5, *p* = *n*.*s*.), as is evident by the non-significant results from a *t test* and an independent 2-group Mann-Whitney *U*-test, respectively. Participants were selected based on specific inclusion and exclusion criteria: (i) participants should not have suffered from dyslexia and other reading difficulties; (ii) they should not have suffered from major depression, ongoing substance abuse, poor vision that cannot be corrected with glasses or contact lenses; (iii) they should not have been diagnosed with other serious psychiatric, neurological or brain-related conditions, such as Parkinson's disease; (iv) they had to be native Swedish speakers; (v) they had to be able to read and understand information about the study; and (vi) they had to be able to give written consent.

**Table 1:** Age and gender of healthy controls (HC) and participants with Mild Cognitive Impairment (MCI).

	***N***		***Age***	
	**F**	**M**	**F**	**M**
HC	19	11	68 (7.6)	69 (5.7)
MCI	13	12	72 (5.1)	70 (5.6)

Healthy controls had a significantly higher Mini-Mental State Exam (MMSE) score. (The MMSE score is a scale of 0–30 and represents the cognitive status of an individual). Mean MMSE score for the MCI participants was 28.2, which is close to *normal* (43). Ethic approvals for the study were obtained by the local ethical committee review board (reference number: L091-99, 1999; T479-11, 2011); while the currently described study was approved by the local ethical committee decision 206-16, 2016.

### 2.3. Acoustic features

#### 2.3.1. Segmentation

**Figure 1:**
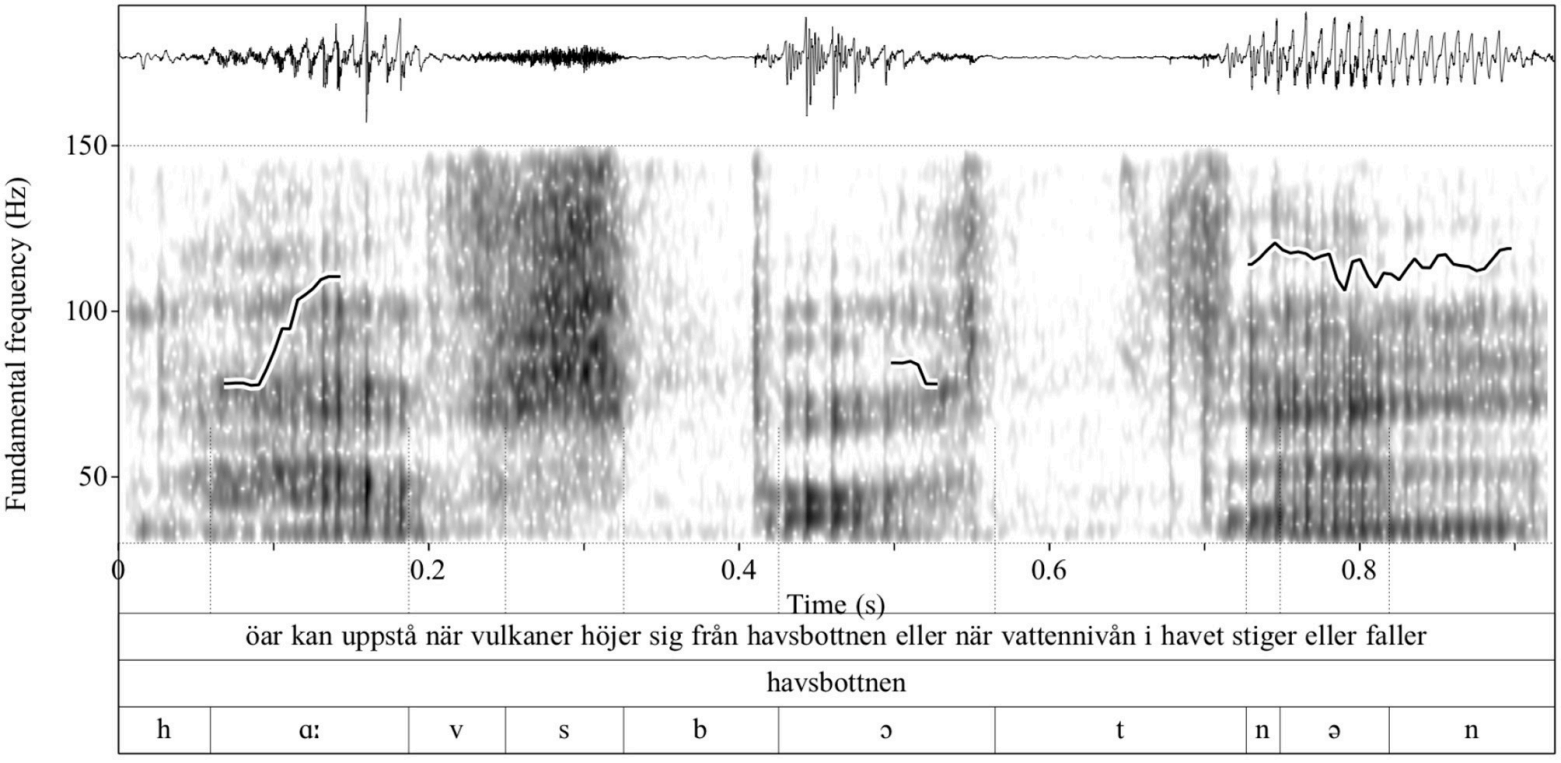
Waveform, spectrogram, and *F*0 contour—superimposed on the spectrogram—of an example utterance (upper tier). Shown in the plot is the segmentation of the word *havsbottnen* “seabed” (middle tier); the individual sounds are shown in the lowest tier. Sound boundaries are indicated with thin vertical lines. The ordinate shows the *F*0 values whereas the abscissa shows the time in second.

Each vowel was segmented in the acoustic signal; that is, we located the right and left boundary of vowels and consonants. A segmentation example is shown in Figure 1. Specifically, the figure shows the waveform (upper panel) and spectogram of the word *havsbottnen* ‘seabed' taken here as an example from a larger sentence: ö*ar kan uppstå när vulkaner höjer sig från havsbottnen eller när vattennivån i havet stiger eller faller* “islands can occur when volcanoes rise from the seabed or when water levels in the ocean rise or fall” (see the Appendix for the whole passage). There are also three different tiers with the transcriptions, the top tier defines the boundaries of sentences; the second tier in the middle shows the word boundaries; and the lower tier shows the segmental boundaries, namely the boundaries of consonants and vowels (see also the thin lines extending from the lower tier to the middle of the spectogram and demarcate vowels and consonants). For the segmentation, we have employed an automatic module for Swedish developed by the first author (44). As measurements and processes rely on accurate segmentation this step is crucial; therefore, all segmentation decisions were evaluated twice based on the following segmentation criteria: vowel onsets and offsets were demarcated by the beginning and end of the first two formant frequencies; the rise of the intensity contour at the beginning of the vowel and its fall at the end of the vowel served as additional criteria for vowel segmentation. Then, we measured the acoustic properties of using Praat (45). Overall, there were 4396 HC and 4273 MCI productions, which is a relatively balanced data set.

#### 2.3.2. Acoustic measurements

Vowel formants were measured at multiple positions. Traditionally vowel formants are measured using a single measurement at the middle of the vowel, which is supposedly the vowel target. Nevertheless, the shape of the formant contour can also convey information about participants' sociophonetic properties (see for a discussion 37). To this end, we conducted three measurements of formants at the 15, 50, 75% of vowels' duration. Vowel formants were calculated using standard Linear Predictive Coding (LPC-analysis) (46). We also measured vowel duration and fundamental frequency (*F*0) (47). The latter is the lowest frequency of speech; and it constitutes the main acoustic correlate of speech melody (a.k.a., intonation) (48). We calculated the minimum, maximum, and mean *F*0 for each vowel. *F*0 and formant frequencies were measured in Hertz.

#### 2.3.3. Sociophonetic features

In addition to the acoustic features, the model included as predictors information about participants' age and gender. Overall, the classification tasks included the following 24 acoustic and sociophonetic predictors:

1. **Vowel Formants:** We measured the first five formant frequencies of vowels (i.e., *F*1, *F*2, *F*3, *F*4, *F*5) at the 15%, 50%, and 75% of the vowels' total duration: i.e., *F*1 15%, *F*1 50%, *F*1 75%…*F*5 15%, *F*5 50%, and *F*5 75%; We also provided the log-transformed values of F1, F2, F3.
2. **Fundamental frequency (***F*0**):** We measured the *F*0 across the duration of the vowel and calculated the *mean F*0, *min F*0, and *max F*0.
3. **Vowel duration:** Vowel duration measured in seconds from vowel onset to vowel offset.
4. **Gender:** Participants' gender.
5. **Age:** Participants' age.

### 2.4. Models and experiments

**Figure 2:**
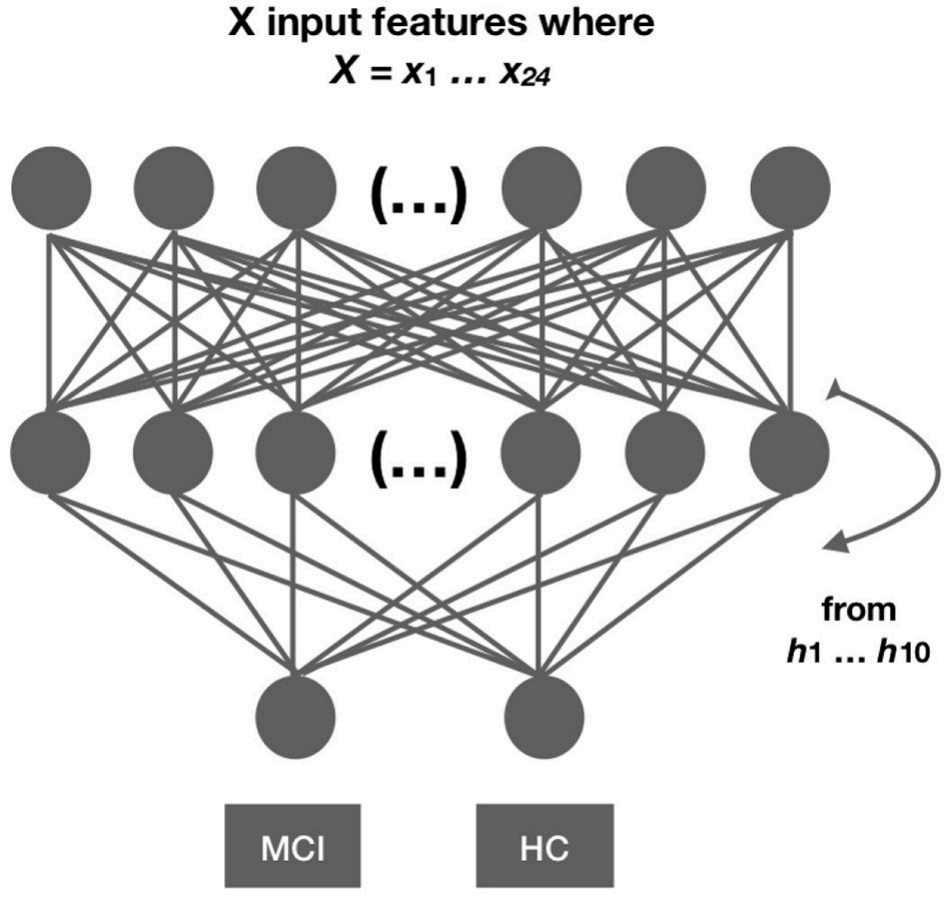
Network architecture. We developed 10 different networks with 21 predictors each. The networks differed in the number of hidden layers ranging from 1…10. Each network architecture was evaluated twice using cross-validation and evaluation split. Model comparison measures are reported for each evaluation separately.

In this section, we describe the neural network architectures employed in this work. Ten neural network architectures that differed in the total number of hidden layers from *h*1…*h*10 were evaluated twice using validation split and cross-validation (the other parameters were the same across models). We present all ten models and not the best model only because (i) we want to demonstrate the whole methodological process that led to the selection of the best model and stress out that the final model is the result of a dynamic process of model comparison; (ii) different randomization of the data may provide different output; thus, a rigorous evaluation can demonstrate whether the output is consistent across models. For example, by demonstrating that the output is not random and that there is a pattern between the different models; and (iii) the evaluation process is being part of the model and not external to the model as it can explain the final architecture of the model, such as the number of hidden layers in the model. An overview of the architectures is shown in Figure 2 and in Table 2. The neural architectures were implemented in Keras, a high-level neural networks API (49) running on top of TensorFlow (50) in Python 3.6.1. For the normalization and scaling of predictors, we employed modules from SCIKIT-LEARN, which is a machine learning library implemented in Python (51, 52).

**Table 2:** Deep neural network architectures with 1 to 10 hidden layers.

**Layer**	**Shape**	**Activation**
Input layer	Dense 300 (21 Input Dimensions)	ReLU
1…10 hidden layers	Dense 300	ReLU
Output layer	1	Sigmoid

*All models employed stochastic gradient descent optimizer with 0.9 Nesterov momentum*.

#### 2.4.1. Model design

1. **Transformation**. All predictors were centered and scaled, using standard scaling, which standardizes the features by removing the mean and by scaling to unit variance (for the scikit-learn implementation of a Standard Scaler, see 52). The mean and standard deviation are estimated on the training set. Then these estimated measures are used to transform the training and test sets separately. So, data in training and test sets are not transformed simultaneously. The reason for conducting different transformations is to avoid a bias from the test features when the mean and the standard deviation are estimated during standard scaling.
2. **Layers**. We tested ten different network architectures that differed in the number of hidden layers from *h*1…*h*10; the input and output layers are excluded. The number of layers in the network can affect its accuracy. Most layers except from the output layer were trained with a ReLU activation function (53, 54). The last layer had a sigmoid activation.
3. **Optimization**. We employed a Nesterov stochastic gradient descent (SGD) optimization algorithm. The learning rate was set to 0.1 and the momentum was set to 0.9.
4. **Epochs and Batch Size**. (a) In cross-validation: network architectures were trained for 80 epochs with 35 as a batch size. (b) In 90%-10% validation split: networks were trained for 100 epochs with 35 as a batch size.

#### 2.4.2. Model comparison and evaluation measures

During the training phase, the neural network learns the acoustic properties that characterize MCI and HC. During the evaluation phase, the network evaluates unknown data vectors from the test set; this time the corresponding label (i.e., MCI or HC) is not available to the model and makes a prediction whether these unknown data vectors correspond to MCI or HC productions. To estimate the performance of the neural network, we compare the predictions of the neural network with the classification made by clinicians using combined imaging and neurological, neuropsychological examination.

A confusion matrix represents the relationship between predicted values and actual values (see Table 3). The columns of Table 3 represent the actual condition (MCI or HC) and the rows represent the positive and negative predictions. A true positive (TP) indicates how many times the condition was MCI and the neural network actually predicted MCI; the false positive (FP) indicates when the condition was HC but the network predicted MCI; the false negative (FN) indicates when the condition was MCI and the network predicted HC; and lastly, the true negative indicates when the condition was HC and the neural network made the correct prediction, namely HC. The different neural network models were compared with each other based on the following evaluation measures: (i) accuracy, (ii) precision, (iii) recall, (iv) F1 score, and (v) ROC/AUC.

1. **Accuracy:** The accuracy is the most commonly employed evaluation measure in classification studies. It refers to the number of correct predictions made by the model divided by the total number of all estimations: *Accuracy* = (*TP*+*TN*)/(*TP*+*TN*+*FP*+*FN*). However, the accuracy is not always the best evaluation measure when the design is unbalanced and corrections are often required. To this end, the precision, recall, *F*1*score*, and ROC/AUC curve provide more balanced estimates.
2. **Precision:** The precision is the number of true positives divided by the sum of true positives and false positives, i.e., *Precision* = *TP*/(*TP*+*FP*). So, when there are many FPs, the precision measure will be low.
3. **Recall:** Recall (a.k.a. sensitivity) is the number of true positives divided by the sum of true positives and false negatives, i.e., *Recall* = *TP*/(*TP*+*FN*). This suggests that a low recall will indicate that there are many FNs.
4. *F*1 *score***:** The *F*1*score* is the weighted average of Precision and Recall: *F*1 *score* = 2 × [(*Precision*×*Recall*)/(*Precision*+*Recall*)]. The *F*1 *score* captures the performance of the models better than the accuracy, especially when the design is unbalanced. A value of 1 indicates a perfect precision and recall, whereas a value of 0 designates the worst precision and recall. Because the *F*1 *score* can be less intuitive than the accuracy, most machine learning studies usually report the accuracy of the model.
5. **ROC/AUC curve:** The receiver operating characteristic (ROC) and the area under the curve (AUC) are two evaluation measures that display the performance of a model. The ROC is a curve that is created by plotting the true positive rate (i.e., the precision) against the false positive rate (i.e., 1-Recall). An optimal model has an ROC closer to 1 whereas a bad model has an ROC closer to 0.

**Table 3:** Confusion matrix.

	**Condition positive**	**Condition negative**
Predicted condition positive	True positive (TP)	False positive (FP)
Predicted condition negative	False negative (FN)	True negative (TN)

#### 2.4.3. Model evaluation

1. **5-fold group cross-validation**. In a “5-fold group cross-validation,” the data are randomized and split into five different folds and the network is trained five times. In each training setting, a different part of the available data is hold out as a test set. The “5 fold group crossvalidation” also ensures that there are no measurements from the same participants in the training and test sets as all data from a given participant will be either in the test set or in the training set but not in both sets (In a simple “5-fold cross-validation” measurements from a given participant might be in both the training and test set after randomization which creates a bias, because the network will be trained on properties from given participants and then asked to provide predictions with respect to these participants). To evaluate the cross-validation, we provide the mean and standard deviation of the accuracy we get from each evaluation. We also provide the ROC curve and the AUC scores that provide a corrected measure of the accuracy.
2. **90–10% Evaluation split**. We also provide the findings from the validation split and discuss in detail validation measures, namely the accuracy of the model, the precision, recall, and *F*1 *score*. To this end, we split the data into two parts. The first part consists of the 90% of the data and functions as a training corpus whereas the second part, the remaining 10% functions as an evaluation set. Just like in the cross-validation, the speakers in the evaluation and test sets are different.

## 3. Results

First, we present the results from the evaluation task and then, we present the results from the validation split.

### 3.1. 5-fold group crossvalidation

**Figure 3:**
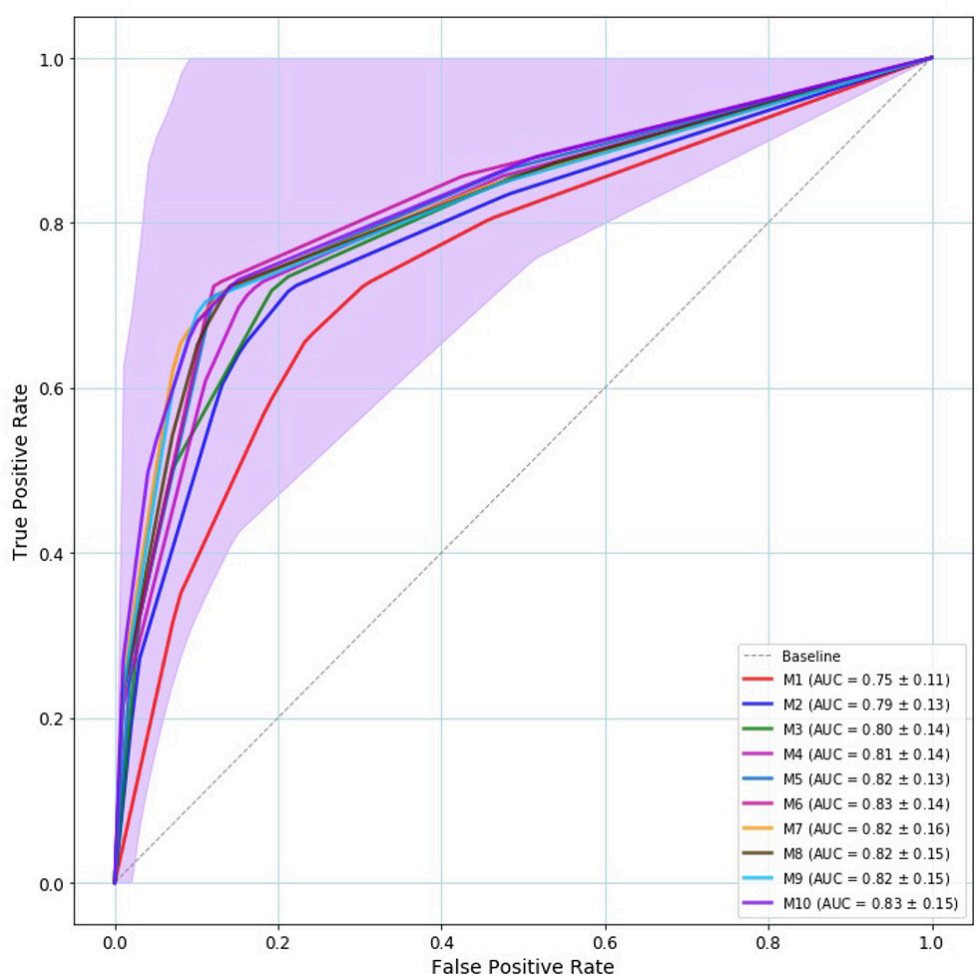
Mean ROC curve and AUC of the 5-fold crossvalidation. Model—*M*1…*M*10— are represented by solid line with a different color. The baseline is represented by a dashed gray line. All models provided ROC curves that were over the baseline. The best model is the model whose ROC curve approaches the left upper corner. The shaded area indicates the M10's *SD* that is the outperforming model both in terms of ROC/AUC (83%) and validation accuracy (83%).

We conducted a 5-fold group cross-validation. Within each fold the model is validated 80 times, which is the number of epochs of the model and the mean accuracy, mean validation accuracy, and the corresponding standard deviation are calculated. Table 4 provides the mean accuracy and the mean validation accuracy along with the corresponding standard deviation that results from the 5-fold crossvalidation. As seen by Table 4 models six to ten are consistent with respect to their classification accuracy. These models have six to ten hidden layers and all resulted in 83% mean cross-validated accuracy. Figure 3 displays the mean ROC curve and AUC of the 10 neural network models. The shaded area indicates the *SD* for the final model: M10. The results from the cross-validation clearly show that when trained using a Sequential Neural Network, speech features can be employed for the identification of MCI. To establish this finding, we provide a second evaluation by training the same networks on the 90% of the data and evaluating on the remaining 10%.

**Table 4:** Model *M*1…*M*10 mean classification accuracy and mean validation accuracy and the corresponding *SD* from the 5-fold crossvalidation.

**Model**	**Accuracy**		**Val. Accuracy**	
	**Mean**	**SD**	**Mean**	**SD**
M1	98	3	75	12
M2	99	3	80	14
M3	99	2	81	15
M4	99	2	82	15
M5	99	2	82	14
M6	99	2	83	15
M7	99	2	83	16
M8	99	2	83	15
M9	99	2	83	16
M10	98	3	83	17

### 3.2. 90–10% evaluation split

Table 5 shows a comparison of the accuracy scores on the training set. The highest accuracy was provided by Model 7 that resulted in 75% classification accuracy and the second best model was Model 5 with 71% classification accuracy at the validation set.

**Table 5:** 90%/10% validation split results.

**Model**	**Accuracy**	**Precision**	**Recall**	**F1 score**
M1	67	86	56	63
M2	68	92	56	66
M3	67	100	49	65
M4	68	63	62	62
M5	71	73	71	71
M6	68	73	72	72
M7	75	100	49	65
M8	65	100	49	65
M9	69	100	49	65
M10	66	95	51	64

*The table shows the accuracy, precision, recall, and f1 score for M1…M10*.

## 4. Discussion

The number of people that are developing dementia is increasing worldwide. Identifying MCI early is of utmost importance as it can enable a timely treatment that can delay its progression. A number of studies have shown that speech and language, which are ubiquitous in everyday communication, can provide early signs of MCI and other prodromal stages of Alzheimer's disease (e.g., 22). The aim of this study has been to provide a classification model for the quick and fast identification of MCI individuals, using data from speech productions.

To this end, we have automatically transcribed, segmented, and acoustically analyzed Swedish vowel productions. The acoustic properties of vowels, namely their formants (*F*1−*F*5), duration, fundamental frequency, age, and gender of participants were employed as predictors. Specifically, ten Deep Neural Networks Architectures were trained on the acoustic productions and evaluated on how well they can identify MCI and healthy individuals, by comparing model predictions (i.e., MCI or HC), with the evaluations conducted by clinicians using combined imaging and neuropsychological examination. We have trained ten models each with a different number of hidden layers. Models 6 to 10 resulted in 83% mean classification accuracy (see Table 4).

One important contribution of this study is that it provides a model that can identify MCI individuals automatically and with high accuracy, providing a quick and early assessment of MCI, by using only a simple acoustic recording, without other neuropsychological or neurophysiological information. Also, it demonstrates that speech acoustic properties play a central role in MCI identification and points to the necessity for more acoustic studies with respect to MCI. Nevertheless, 83% accuracy might still be low for clinical use, if it is going to be employed as the only assessment. Two aspects can account for these accuracy results. First, there is a significant symptom variability among individuals with MCI, which has been stressed out by a number of papers including consensus papers for the diagnosis of MCI (e.g., 4, 2). Some of these symptoms are not related to speech, thus additional phonemic, moprhosytactic, etc., predictors might increase the accuracy. Also, by increasing the data and retraining the model, it is possible to improve model accuracy as it is evidenced by the fact that some of the crossvalidation folds resulted in considerably higher accuracy (cf., the SD is between 14 and 17%).

Moreover, this study presents the methodological process that can lead to the selection of the classification model of MCI vs. HC and the evaluation techniques that enable the selection of the final model from a set of ten different models. We have discussed two methods: i. validation split, and ii. crossvalidation. In the validation split, model 7 resulted in the highest accuracy, namely 75%. Nevertheless, the validation split is a weak evaluation method as it depends on the data selected as a training set and as a test set; different randomization of the data may provide a different output. It also depends on the split size (e.g., 75–25%, 80–20%, 90–10%). To avoid these confounds, we conducted a 5-fold crossvalidation, which performs multiple splits of the data, depending on the number of validation folds (cf. 55, 56). Most importantly, the significance of the proposed machine learning model formulation is not that it provides a specific model only but also because it offers a process for continuous evaluation and improvement of the model. Therefore, model evaluation and model comparison constitute indispensable parts of machine learning.

Future research is required (i) to evaluate multivariable acoustic predictors, e.g., predictors from consonants and non-acoustic predictors, i.e., linguistic features, such as parts of speech, syntactic and semantic predictors, sociolinguistic predictors like the education of the speaker; (ii) to establish whether these acoustic variables could be useful in predicting conversion from MCI to dementia; and (iii) to create an automated differential diagnostic tools, which will enable the classification of unknown MCI individuals from conditions with similar symptoms (cf., 57). A system of this form, will require more data from a larger population, yet our current findings do provide a promising step toward this purpose.

In conclusion, this study has showed that a Deep Neural Network architecture can identify MCI speakers and can potentially enable the development of valid tools for identifying cognitive changes early and enable multidomain life style interventions and/or pharmacological treatments at the MCI stage, which can potentially delay or even prevent the development of AD and other types of dementia.

## Author contributions

CT conducted the acoustic analysis of the materials, designed and run the Deep Neural Networks architectures and wrote the first draft of the paper. DK supervised the data collection. Subsequently all authors worked on refining and revising the text. All authors approved the final version.

### Conflict of interest statement

The authors declare that the research was conducted in the absence of any commercial or financial relationships that could be construed as a potential conflict of interest.

## Appendix

- **Text in Swedish:** Ordet ö beskriver ett område som är helt avskuret från land och som är omgivet av vatten på alla sidor. Öar kan uppstå när vulkaner höjer sig från havsbottnen eller när vattennivån i havet stiger eller faller. Ett flertal öar uppstod mot slutet av den förra istiden. När isen smälte och vattnet rann ut i havet höjdes vattennivån så mycket att de låga landområdena översvämmades. Idag ser man bara de högsta topparna sticka upp över vattenytan som öar. Djur och växter som på något sätt lyckas ta sig till en avlägsen ö kan sedan vanligtvis inte komma därifrån igen. För att överleva är de därför tvungna att mycket snabbt anpassa sig till den nya omgivningen. De levande arter som finns på öar löper en ständig risk att bli utrotade. Detta kan inträffa när nya djur dyker upp eller när människor kommer dit och börjar störa dem.
- **Translated text in English:** The word island describes an area that is completely cut off from the land and is surrounded by water on all sides. Islands can occur when volcanoes rise from the seabed or when water levels in the ocean rise or fall. A number of islands occurred at the end of the last ice age. When the ice melted and the water ran out into the sea, the water level was raised so much that the low lands were flooded. Today, only the highest peaks can be seen across the water surface as islands. Animals and plants that somehow manage to get to a distant island usually do not leave the place again. Therefore, in order to survive, they are forced to adapt very quickly to the new environment. The living species on islands run a constant risk of being extinct. This can happen when new animals appear or when people get there and start to disturb them.
